# A case of pulmonary inflammatory myofibroblastic tumor treated with bronchoscopic therapy plus lobectomy

**DOI:** 10.1186/s13019-021-01528-5

**Published:** 2021-05-26

**Authors:** Fan Yang, Wenxia Zhang, Cheng Han, Hanliang Jiang

**Affiliations:** Regional Medical Center for National Institute of Respiratory Diseases, Sir Run Run Shaw Hospital, School of Medicine, Zhejiang University, 3 Qingchun East Road, Jianggan District, Hangzhou, 310016 China

**Keywords:** Inflammatory myofibroblastic tumor, Lung neoplasm, Atelectasis, Interventional bronchoscopy, Surgery and oncology

## Abstract

**Background:**

Inflammatory myofibroblastic tumor (IMT) is a rare tumor with malignant potential. We presented a case of a young adult who was diagnosed with IMT and treated with loop electrocautery therapy to relieve airway obstruction, followed by lobectomy to complete resection. Recent studies have supported the use of such interventional resection methods.

**Case presentation:**

A non-smoking 30-year-old woman presented with a 1-month history of progressive dyspnea and productive cough. The Chest X-ray showed a homogenous opacity invading the entire left hemithorax, and the mediastinum content was attracted to the left side. In an effort to avoid pneumonectomy and afford rapid palliation of dyspnea, loop electrocautery was selected as the most appropriate therapy. The left upper lobectomy by thoracoscopy was performed instead of left upper lobe sleeve resection in order to better prevent the recurrence of lung atelectasis. After 6 years of follow-up, no evidence of recurrence has been found till now.

**Conclusion:**

Interventional bronchoscopy coupled with surgical resection serves not only as a palliative management to bronchial obstruction but also a way to avoid pneumonectomy.

## Introduction

Inflammatory myofibroblastic tumor (IMT), first described by Brunn in 1939 [1], is a rare disease entity [2], also known as plasma cell granuloma or inflammatory pseudotumor. So far, it is still not clear whether these lesions are primary inflammatory processes or low-grade malignancies with obvious inflammatory reactions. Diagnosis of IMT is difficult to establish before surgery due to its diversified radiologic manifestations [3, 4], as the tumor can be cystic or homogeneous, parenchymal or endobronchial [5]. Complete surgical resection is a treatment option not only applied to exclude malignancy but also to achieve a good prognosis [6, 7]. In this report, we presented a case of IMT which was successfully removed by interventional bronchoscopy plus lobectomy [8].

## Case presentation

**Fig. 1:**
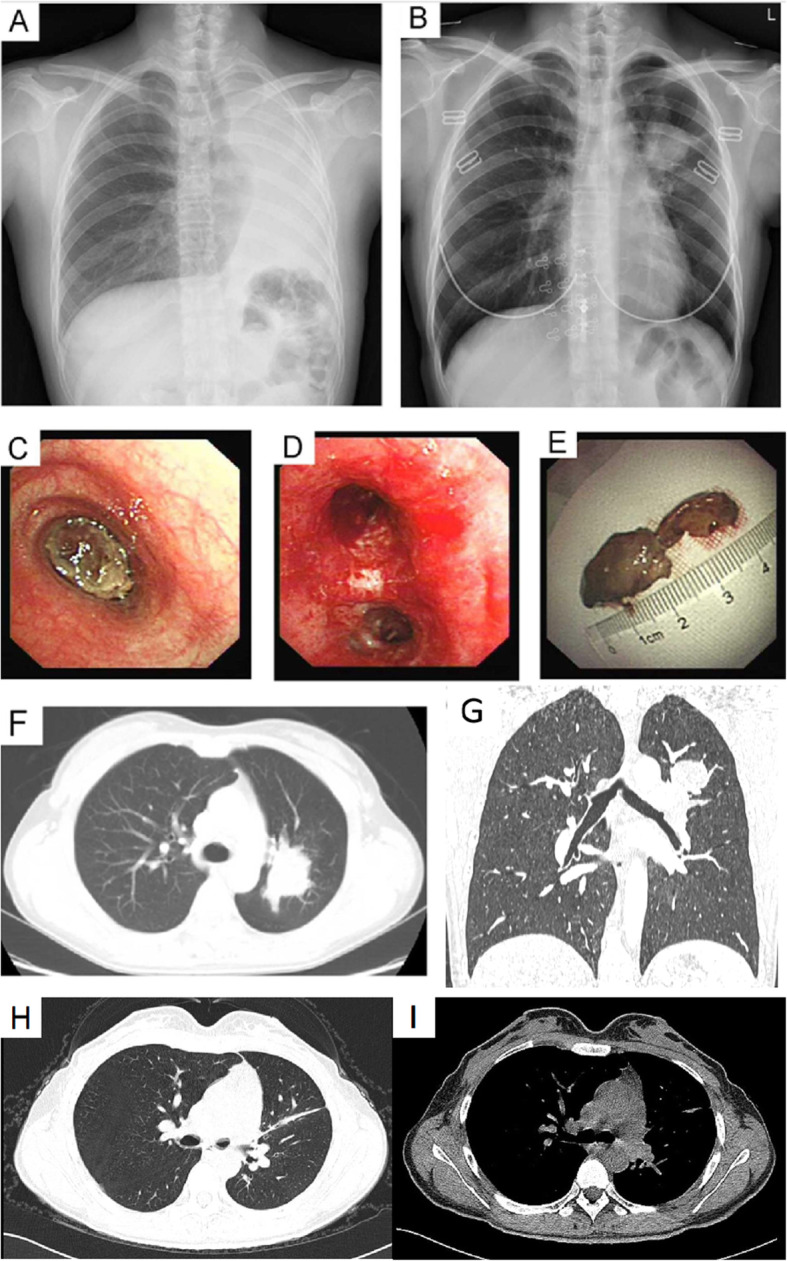
Pulmonary mass. **a**: Left lung atelectasis was established by chest X-ray test. **b**: Chest X-ray showed a re-expansion of the left lung and a mass in the left upper lobe after interventional therapy. **c**: Bronchoscopy showed a tumor that completely obstructed the left main bronchus. **d**: The interventional therapy fully re-opened the obstructed left main bronchus and the lower subsegment of the left lower lobe. **e**: The lesion was partially resected by loop electrocautery via flexible bronchoscopy. **f**: Chest CT scan showed a tumor on the left upper lobe. **g**: Coronal CT after loop endoscopically resection on the left upper lobe. **h**: Chest CT scan showed the residual scar tissue after surgery a week later. **i**: CT demonstrated no relapse after a 6-year follow-up in 2019.12

A non-smoking 30-year-old woman presented with a 1-month history of progressive dyspnea and productive cough. Her Chest X-ray showed a homogenous opacity invading the entire left hemithorax, and the mediastinum content was attracted to the left side. Hence, a diagnosis of left lung atelectasis was established (Fig. 1a). Bronchoscopy showed that the tumor completely obstructed the left main bronchus (Fig. 1c).

In an effort to avoid pneumonectomy and afford rapid palliation of dyspnea, loop electrocautery was selected as the most appropriate therapy. The lesion was partially resected by loop electrocautery under the guidance of a flexible bronchoscopy [9]. Following resection, plenty of sputum was sprayed from the left lower lobe. This intervention completely re-opened the obstructed left main bronchus and the lower subsegment of the left lower lobe (Fig. 1d). Thereupon, the patient’s dyspnea was resolved. Nevertheless, chest X-ray showed a re-expansion of the left lung and a mass on the left upper lobe (Fig. 1b). After bronchoscopic procedure, the chest computed tomography (CT) scan showed a tumor on the left upper lobe (Fig. 1f and g).

Histologic examination of the lesion indicated inflammatory granulation tissue, the immunohistochemical (IHC) examination showed: CK (−); EMA (−); Vimentin (+); SMA (+); Desmin (−); Calponin (−); ALK (−); S-100 (−); CD34 (−); Ki67 (+); EBER (−), and thus the pathological diagnosis was IMT.

**Fig. 2:**
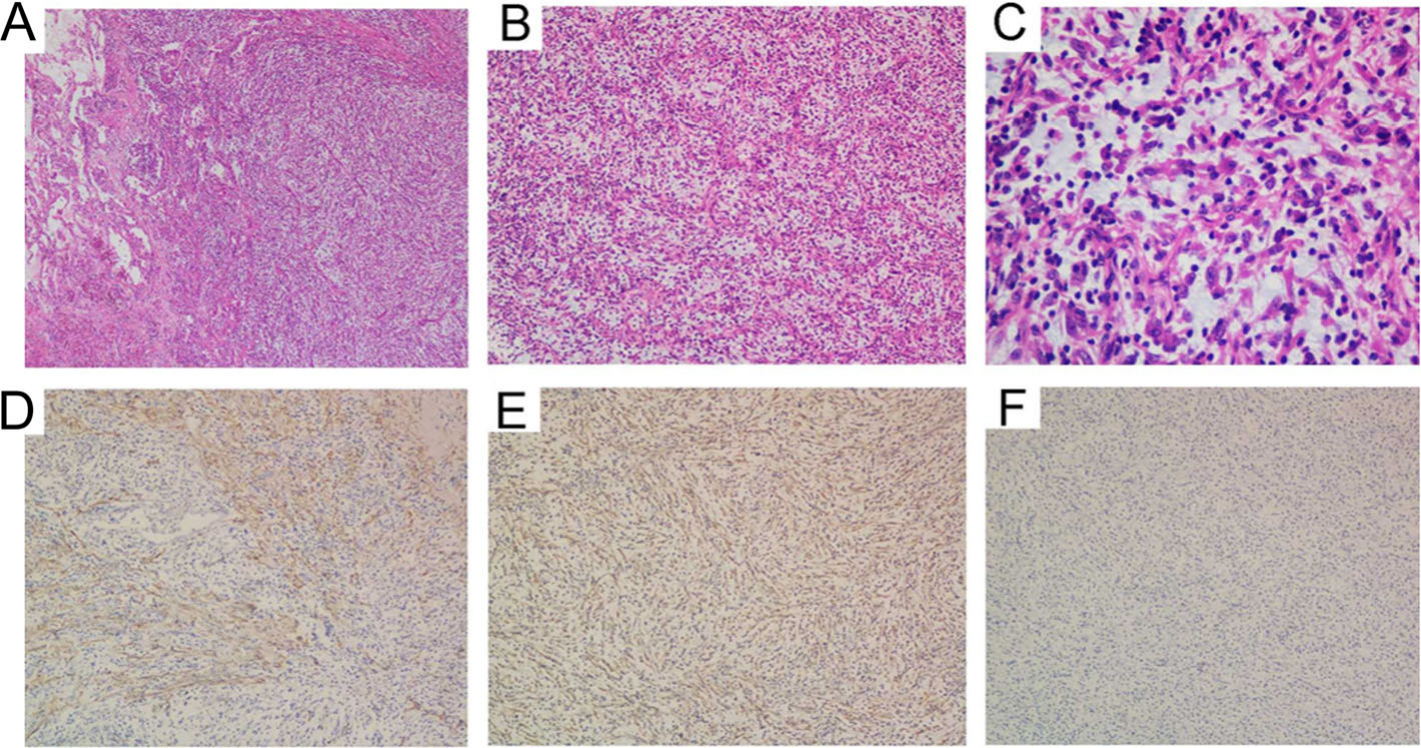
Inflammatory myofibroblastic tumor. **a**: Low power view demonstrating intersecting fascicles of spindle cells in lung tissue associated with abundant vessels. **b**: Note abundant blood vessels in a myxoid stroma with loosened spindle cells, scattered lymphocytes and plasma cells. **c**: High power view showed spindle tumor cells with eosinophilic cytoplasm lack prominent nuclear atypia, though mitoses could be found. Tumour cells showed immunoreactivity for **d**: smooth muscle actin; **e**: Vimentin; and negativity for **f**: ALK

Thus, the surgery was resorted for diagnostic and therapeutic purposes after 45 days, consisting of a left upper lobectomy by thoracoscopy. We sent the specimen for frozen section and the pathological diagnosis was Inflammatory granulation tissue hyperplasia. Under gross examination, the tumor was 5 cm in size, and it was soft, jelly-like, homogeneous and invaded the bronchial wall locally. Besides, microscopic examination revealed proliferative regular spindle cells arrayed in fascicles, as well as admixed with lymphocytes, plasma cells and eosinophils (Fig. 2a, b and c). IHC examination revealed: Vimentin (+); SMA (+); CK (−); Myogenin (−); Desmin (−); S100 (−); CD117 (−); ALK (−) (Fig. 2d, e and f). No histologic evidence of metastasis to the lymph tissue was observed (0/8) and the bronchial resection margin was negative. Only the residual scar tissue was found in CT after the surgery (Fig. 1h). Based on these data, the diagnosis of IMT was established. The patient was followed up every 3 months and received CT scan every 6 months. After 1 year of follow-up, no evidence of recurrence was found. The patient was lost to follow-up in the past 6 years until 8 months ago in 2019.12. Meanwhile the CT demonstrated no recurrence in the past 6 years (Fig. 1i).

## Discussion

IMT is a rare pathologic entity, with its morbidity among patients with lung resection about 0.04% [10]. IMT can occur in all age groups, but more than half of the patients are younger than 40 years old, meanwhile, it is indeed one of the most common primary lung tumors in the pediatric age group [11]. Most patients have respiratory symptoms, such as cough, dyspnea, fever, fatigue, and hemoptysis, whereas about 70% of patients have no symptoms [12]. Airway obstruction caused by IMT is rare [13]. The endobronchial and invasive parenchymal mass can easily cause pulmonary symptoms [14].

The radiologic features of IMT have been analyzed by Agrons [15] and the conclusion was that the radiological features are variable and non-specific. Infiltration of airways, calcification, and cavitation occur rarely. Most of the IMT is located peripherally and most of the time in the lower lobes. We presented a case of uncommon IMT which was in the central and affecting upper lobe, a similar report has been described before [16] coincidentally.

Diagnosis of IMT is dependent on surgical specimens. Because of the difficulty in distinguishing it from malignant tumors in small tissue samples that obtained from bronchoscopic examination, the diagnosis of IMT can also produce false positive and false negative results even with partially successful fine-needle aspiration biopsy [17]. Similarly, bronchoscopy can also hardly succeed as endobronchial IMT accounts for less than 5% [18].

For patients who are not suitable for complete surgical resection, glucocorticoids, radiotherapy and chemotherapy can be supplied, yet both treatment success and treatment failure can be achieved [10]. In this report, we presented an extraordinary case where the patient won an opportunity for operation by receiving an interventional bronchoscopy treatment. It was essential and requisite to the patient as the tumor was found to be derived from the left upper lobe after loop electrocautery via flexible bronchoscopy. Therefore, the surgeon adopted left upper lobectomy instead of left pneumonectomy. This approach not only served as a palliative management to bronchial obstruction, but also a way to avoid pneumonectomy [8]. Finally, the surgery performed was ordinary left upper lobectomy instead of left upper lobe sleeve resection in order to better prevent the recurrence of lung atelectasis. It is expected to be valuable for patients with similar medical conditions to undergo an interventional bronchoscopy before surgical resection.

The prognosis of patients undergoing complete resection of IMT is known to be with 5-year and 10-year survival rate of 91 and 77% [10]. The recurrence of IMT is rare, but it can be up to 60% in cases of incomplete resection [10] and the vast majority of deaths are secondary to distal metastases [19]. Finally, the surgery performed was ordinary left upper lobectomy instead of left upper lobe sleeve resection in order to better prevent the recurrence of lung atelectasis in this report. Postoperative pathology suggested that tumor infiltrating the bronchus could better prove this view. The patient was followedup regularly in the first year after the surgery and lost to follow-up in the past 6 years until 8 months ago. Fortunately, there was no evidence of relapse after the examination. Therefore, we proposed a follow up protocol for IMT: the CT should be received 3 months at the first 2 years after resection and extend to 6 months in the following 3 years. Annual follow-up should be finished in the last 5 years and the follow-up time should last for 10 years compulsively.

In all, IMT is rare, yet it should be considered as a differential diagnosis of pulmonary lesions. The prognosis of IMT is highly dependent on complete surgical resection. Besides, interventional bronchoscopy coupled with surgical resection can help avoid unnecessary pneumonectomy.

## Data Availability

The data used to support the finding of this study are included within the article. The data and materials in the current study are available from the corresponding author on reasonable request.

## Abbreviations

IMT: Inflammatory myofibroblastic tumor
CT: Computed tomography
IHC: Immunohistochemical
